# Liquid-fermentation-derived *Candida utilis* protein improves nutrient digestibility and intestinal health in weaned piglets

**DOI:** 10.3389/fvets.2026.1862132

**Published:** 2026-06-30

**Authors:** Shiqiao Wu, Junqing Guo, Xiaohong Wang, Wenqiang Guo, Ruqing Zhong, Bao Yi, Miao Li, Gengcan Li, Yue Hao, Liang Chen, Hongfu Zhang

**Affiliations:** 1 State Key Laboratory of Animal Nutrition and Feeding, Key Laboratory of Animal Nutrition and Feed Science of Ministry of Agriculture and Rural Affairs, Institute of Animal Science, Chinese Academy of Agricultural Sciences, Beijing, China; 2 College of Animal Science, Inner Mongolia Agricultural University, Hohhot, China; 3 Inner Mongolia Hailin Technology Development Co., Ltd., Tongliao, China; 4 National Data Center of Agricultural Environment, Beijing, China

**Keywords:** *Candida utilis* single-cell protein, gut microbiota, intestinal health, nutrient digestibility, soybean meal replacement, weaned piglets

## Abstract

**Objective:**

This study aims to elucidate the nutritional functions and practical application value of *Candida utilis* single-cell protein derived from corn steep liquor in piglet diets.

**Methods:**

In this study, 90 weaned piglets (initial body weight: 7.24 ± 1.30 kg) were randomly assigned to 3 dietary treatments for 28 days, with soybean meal partially replaced by 0, 3%, or 5% corn steep liquor-derived *Candida utilis* single-cell protein (FP50) in diets formulated on an iso-nitrogenous and iso-energetic basis.

**Results:**

Dietary inclusion of FP50 did not impair growth performance, feed efficiency, or diarrhea incidence. In contrast, apparent total tract digestibility of DM and CP increased with increasing FP50 inclusion (*p* < 0.01). Intestinal status was improved, as evidenced by reduced jejunal CD (*p* < 0.05), increased V/C ratio (*p* < 0.05), and decreased concentrations of BCFAs in the cecum (*p* < 0.05). Moreover, FP50 supplementation modulated the cecal microbiota by enriching specific genera (e.g., *Blautia*, *Romboutsia*, and *Anaerobutyricum*), which Spearman correlation analysis revealed were positively associated with nutrient digestibility and intestinal morphology, but negatively associated with cecal BCFAs. Additionally, FP50 enhanced systemic antioxidant capacity, as reflected by increased serum SOD activity (*p* < 0.05).

**Conclusion:**

FP50 can partially replace soybean meal in weaned piglet diets without compromising growth performance, while improving nutrient utilization and intestinal health. Among the tested levels, 5% substitution exhibited the most consistent improvements, indicating that low-level FP50 inclusion may represent a feasible strategy for reducing soybean meal use in weaned piglet diets.

## Introduction

Global food security is facing increasing challenges due to population growth, climate change, and geopolitical tensions affecting agricultural trade. Within this context, the intensive livestock sector’s heavy reliance on soybean meal—the predominant protein source in monogastric animal diets—has emerged as a critical vulnerability (1). Soybean production is often accompanied by excessive consumption of natural resources and environmental pollution (2, 3). Consequently, developing alternative protein sources to partially replace soybean meal in animal feed has become a strategic priority for sustainable livestock production, particularly in countries that rely heavily on imported soybean resources (4).

The weaning period represents a critical window in pig production, characterized by immature digestive physiology, underdeveloped immune function, and heightened susceptibility to dietary stressors (5). Piglets during this phase are highly sensitive to the source and quality of dietary protein. Soybean meal, despite its favorable amino acid profile, contains various anti-nutritional factors, including antigenic proteins (glycinin and *β*-conglycinin) and trypsin inhibitors, which can trigger transient hypersensitivity, intestinal inflammation, villus atrophy, and post-weaning diarrhea (6, 7). Thus, alternative protein sources are needed that provide adequate nutrition while remaining safe and well tolerated by the immature gut. Single-cell protein (SCP), produced through microbial fermentation of various organic substrates (8, 9), is a high-quality protein resource. Among various SCP-producing strains, *Candida utilis* (*C. utilis*) has garnered considerable attention due to its high protein content and rich nutrient profile (10), rapid growth rate, and ability to utilize low-cost industrial by-products (such as corn steep liquor) for fermentation (11–13), making it a promising candidate for replacing traditional protein sources. Liquid fermentation technology further enhances the nutritional quality of yeast-derived SCP by increasing the proportion of soluble proteins and small peptides, which may facilitate enzymatic hydrolysis and nutrient absorption in monogastric animals while reducing potential anti-nutritional components (14). Currently, research on *C. utilis* SCP in animal nutrition has progressively shifted from “whether it can replace soybean meal” to “how to replace it safely and efficiently” (15, 16). However, most previous studies have focused on high-proportion (≥10%) soybean meal replacement (17, 18), or have evaluated *C. utilis* primarily as a feed additive (15, 19). In contrast, the low-level substitution range (3–5%), which may be more practical for commercial feed formulation, has rarely been systematically evaluated. Furthermore, the specific nutritional effects of *C. utilis* SCP derived from corn steep liquor via liquid fermentation remain poorly defined in weaned piglets.

Beyond the practical inclusion levels, the underlying biological mechanism through which this specific SCP product modulates intestinal homeostasis warrants investigation. Liquid fermentation technology yields a high proportion of soluble proteins and small peptides, which are expected to be highly digestible. Therefore, we hypothesized that the high solubility of FP50 would enhance nutrient disappearance in the foregut, thereby limiting the substrate available for harmful proteolytic fermentation in the hindgut. This shift in the fermentation profile would, in turn, promote better intestinal morphology and microbial balance.

To test this hypothesis, the present study replaced soybean meal with 0, 3, and 5% of FP50 in the diets of weaned piglets, respectively. By integrating data such as nutrient digestibility, intestinal morphology, and microbiota analysis, we aimed to clarify the nutritional functionality and practical applicability of this corn steep liquor-derived *C. utilis* SCP in piglet diets.

## Materials and methods

### Animal ethics statement

The animal experiment was approved by the ethical committee of Institute of Animal Science, Chinese Academy of Agricultural Sciences, CAAS, Beijing (Inspection code IAS2025-110).

### Experimental raw materials

The *C. utilis* SCP (FP50) used in this study was provided by Inner Mongolia Hailin Technology Development Co., Ltd. This product is produced using a three-stage liquid fermentation process with *C. utilis*, utilizing corn steep liquor and glucose as substrates, followed by enzymatic hydrolysis, concentration via a low-temperature evaporator, and finally spray granulation in a fluidized bed. Compared with traditional solid-state fermented yeast products, this process significantly improves the proportion of soluble protein and small molecular peptides, reduces anti-nutritional factors in yeast cell wall, and achieves high-value utilization of agricultural and industrial by-products (20). The product contains approximately 50.93% crude protein and 45.32% total amino acids, and is rich in threonine. The major nutritional composition and amino acid profile of FP50 are presented in Table 1. The mycotoxin content also meets the standards (Supplementary Table S1). *In vitro* nutrient digestibility of FP50 was evaluated using a monogastric animal bionic digestion system, following the *Operation Manual for the Simulated Digestion System of Monogastric Animals* (21), and the results are presented in Supplementary Table S2.

**Table 1:** Chemical composition and amino acid profile of FP50 (%, as-fed basis).

Items	Content
DM	94.43
CP	50.93
EE^1^	0.00
Ash	18.21
CF^2^	0.00
Ca	0.11
β-Glucan	3.26
Mannan	1.59
Amino acids, %	
Aspartic acid	3.26
Threonine	2.00
Serine	2.29
Glutamic acid	8.67
Proline	4.15
Glycine	2.61
Alanine	4.33
Valine	2.43
Isoleucine	1.47
Leucine	3.74
Tyrosine	1.17
Phenylalanine	1.39
Histidine	1.51
Lysine	2.01
Arginine	2.03
Tryptophan	0.26
Cystine	1.20
Methionine	0.80
Total amino acids	45.32

DM, dry matter; CP, crude protein; EE, ether extract; CF, crude fiber.

^1,2^EE and CF contents were below the detection limits of the analytical methods.

### Experimental design and management

A total of 90 castrated male piglets (Duroc × Landrace × Yorkshire), with an initial body weight of 7.24 ± 1.30 kg, were used in this study. The piglets were assigned to three dietary treatments based on initial body weight, with 6 pens per treatment and 5 piglets per pen. A corn-soybean meal basal diet was used. The three treatment diets were formulated based on the principles of iso-nitrogenous and iso-energetic balance, replacing soybean meal with 0, 3, and 5% FP50, respectively. The experiment lasted for 28 days. Dietary formulations followed the nutrient recommendations for castrated pigs (22). The detailed diet composition and nutrient levels are presented in Table 2.

**Table 2:** Ingredients composition and analyzed nutrient composition of the experimental diets (as-feed basis).

Items	FP50 levels, %		
	0	3	5
Ingredients, %			
Corn	36.865	36.87	36.785
Broken rice	10.00	10.00	10.00
Fermented soybean meal	3.00	3.00	3.00
Extruded corn	11.00	11.00	11.00
Soybean meal (46% CP)	15.00	12.00	10.00
FP50	0.00	3.00	5.00
Extruded soybean	10.00	10.00	10.00
Montmorillonite	0.20	0.20	0.20
NaCl	0.40	0.40	0.40
Fish meal	3.00	3.00	3.00
Sucrose	2.50	2.50	2.50
Dextrose	2.50	2.50	2.50
Ca(HCOO)_2_	1.40	1.40	1.50
Ca(H_2_PO_4_)_2_	1.30	1.30	1.30
L-Lysine (98.5%)	0.54	0.56	0.56
DL-Methionine (98.5%)	0.08	0.07	0.07
L-Threonine	0.185	0.17	0.15
L-Tryptophan	0.03	0.03	0.035
Premix^1^	2.00	2.00	2.00
Total	100.00	100.00	100.00
Nutrient levels^2^			
Net energy, Mcal/kg	2.38	2.37	2.36
Dry matter, %	90.42	90.75	90.85
Crude protein, %	19.29	19.43	19.33
Ether extract, %	3.80	3.77	3.71
Crude fiber, %	2.34	2.20	2.23
Ash, %	2.83	3.23	3.28
Calcium, %	0.82	0.82	0.86
Total phosphorus, %	0.67	0.75	0.76
Available phosphorus, %	0.48	0.48	0.48
Lysine, %	1.35	1.35	1.35
Methionine, %	0.39	0.39	0.39
Threonine, %	0.79	0.79	0.79
Tryptophan, %	0.22	0.22	0.22

1 Provided per kilogram of diet: Vitamin A 15000 IU; Vitamin B₁ 4.32 mg; Vitamin B₂ 12 mg; Vitamin B₆ 4.86 mg; Vitamin B₁₂ 30 μg; Vitamin C 180 mg; Vitamin D₃ 4,500 IU; Vitamin E 72.5 mg; Vitamin K₃ 4.5 mg; Nicotinamide 41.58 mg; Calcium pantothenate 19.32 mg; Folic acid 1.764 mg; Biotin 480 μg; Choline chloride 880 mg; Cu 15 mg; Fe 145.5 mg; Zn 1,551 mg; Mn 49.6 mg; Co 0.2 mg; I 0.6 mg; Se 0.4 mg.

2 Nutrient levels are calculated values unless otherwise stated.

During the experimental period, all piglets were housed in a temperature-controlled room with plastic slatted floors. Each pen (1.5 × 2.0 m) was equipped with a stainless-steel feeder and a nipple drinker to provide ad libitum access to feed and water. The ambient temperature was maintained at 28 °C during the first week and decreased by 1.5 °C each week thereafter. The relative humidity was kept at 60–70%. A 7-day adaptation period was provided before the formal trial began. On day 1 and day 28, piglets in each replicate were fasted for 12 h before being weighed to determine the average daily gain (ADG). Feed intake was recorded daily for each replicate. From these data, average daily feed intake (ADFI) and feed-to-gain ratio (F/G) were calculated for each group. Additionally, fecal consistency was observed and scored daily at 08:00 and 16:00 using the established scoring system to record diarrhea incidence and calculate the diarrhea rate (23).

### Sample collection and preparation

On days 26 to 28 of the experiment, fresh fecal samples were collected at 09:00, 14:00, and 17:00. To minimize contamination, disposable gloves were changed between piglets, and samples were immediately transferred into sterile containers. After collection, 10 mL of 10% (v/v) H₂SO₄ was added to each fecal sample to acidify the material and minimize nitrogen loss caused by ammonia volatilization during storage and processing (24, 25). The samples were then stored at −20 °C until analysis. After the trial ended, fecal samples collected from each replicate over the three-day period were pooled and homogenized. Approximately 100 g of the homogenized sample was placed in a 65 °C forced-air drying oven for 48 h. After drying, the sample was equilibrated at room temperature for 24 h, ground to pass through a 40-mesh sieve, sealed, and stored at −20 °C for subsequent determination of nutrient digestibility.

On day 28, after the conclusion of the trial, one pig with a body weight closest to the replicate’s average body weight was selected from each replicate for blood sampling and subsequent slaughter. Blood samples (approximately 10 mL) were collected from the anterior vena cava into vacuum tubes without anticoagulant. After standing at room temperature for 30 min, the samples were centrifuged at 1006 × *g* for 10 min to obtain the serum. The serum was transferred into 1.5 mL microcentrifuge tubes and stored at −80 °C for subsequent analysis. Subsequently, one pig with a body weight closest to the replicate’s average was selected from each replicate for slaughter. Various intestinal segments were promptly dissected. A 1–2 cm segment of jejunum was collected and fixed in 10% neutral buffered formalin for intestinal morphological analysis. Cecal digesta was collected, snap-frozen in liquid nitrogen, and stored at −80 °C for the determination of volatile fatty acid concentrations and microbiological analysis.

### Chemical analysis

The nutritional composition of the experimental diets was determined according to the standard methods of the Association of Official Analytical Chemists (AOAC) as follows: dry matter (DM; AOAC 930.15), crude protein (CP; AOAC 984.13), ether extract (EE; AOAC 920.39), crude fiber (CF; AOAC 978.10), ash (AOAC 942.05), calcium (Ca; AOAC 984.27), total phosphorus (P; AOAC 965.17), and amino acids (AOAC 994.12). The net energy (NE) value was calculated from ingredient composition using established prediction equations (22).

The apparent total tract digestibility (ATTD) of nutrients, including DM, EE, CP, and gross energy (GE), was determined using the endogenous indicator method (acid insoluble ash, AIA (26)). The calculation formula is as follows:

$$D\;(\%)=100–100\times [(A_{2}/A_{1})\times (B_{1}/B_{2})].$$

Where: D is the apparent digestibility (%) of a specific nutrient. A₁ is the content (%) of that nutrient in the diet. A₂ is the content (%) of that nutrient in the feces. B₁ is the AIA content (%) in the diet. B₂ is the AIA content (%) in the feces.

### Serum biochemical parameters

Serum biochemical parameters, including total protein (TP), albumin (ALB), total cholesterol (TC), triglycerides (TG), high-density lipoprotein cholesterol (HDL-C), low-density lipoprotein cholesterol (LDL-C), urea, creatinine (CREA), glucose (GLU), aspartate aminotransferase (AST), alanine aminotransferase (ALT), and alkaline phosphatase (ALP), were measured using a Mindray BS-420 automatic biochemistry analyzer (Shenzhen Mindray Bio-Medical Electronics Co., Ltd., China). The levels of immunoglobulin A (IgA), immunoglobulin G (IgG), immunoglobulin M (IgM), and secretory immunoglobulin A (SIgA) were determined by ELISA with commercial kits (Jiangsu Sumaike Biotechnology Co., Ltd., China). The activities of superoxide dismutase (SOD), glutathione peroxidase (GSH-Px), and catalase (CAT), together with malondialdehyde (MDA) content and total antioxidant capacity (T-AOC), were assessed using commercial antioxidant assay kits from Beijing Huaying Institute of Biotechnology, China. All assays followed the manufacturers’ instructions.

### Histomorphological analysis

The fixed intestinal segments were dehydrated with alcohol, cleared with xylene, embedded in paraffin, and cut into 6 μm sections. Sections were stained with hematoxylin and eosin (HE) and examined under a light microscope. For each slice, at least five intact and clearly visible villus − crypt units were selected. Images were captured using Image-Pro Plus 6.0 software (version 6; Media Cybernetics, Rockville, MD, USA), and villus height (VH) and crypt depth (CD) were measured. The villus height to crypt depth ratio (V/C) was then calculated.

### Volatile fatty acids

The cecal digesta were thawed and homogenized at 4 °C. Approximately, 1.0 g of the sample was aseptically weighed into a 10 mL centrifuge tube, and ultrapure water was added to bring the total volume to 8 mL. After thoroughly disrupting the digesta, the mixture was sonicated for 20 min, followed by centrifugation at 10,000 × *g* for 15 min at 4 °C. The supernatant was collected and diluted 100-fold with ultrapure water. A 1.5 mL aliquot of the diluted solution was withdrawn with a syringe, filtered into a dedicated vial, and analyzed for volatile fatty acids (VFAs) content using an ion chromatography system (Ionpac AS11, USA).

### Microbial analysis

Cecal digesta stored at −80 °C were thawed, and microbial genomic DNA was extracted. DNA integrity was confirmed by agarose gel electrophoresis. The V3 -V4 region of the 16S rRNA gene was amplified with barcoded primers 341F (5′-CCTACGGGNGGCWGCAG-3′) and 806R (5′-GGACTACHVGGGTATCTAAT-3′). High-throughput sequencing was carried out on an Illumina NovaSeq 6,000 platform by Shanghai Majorbio Bio-pharm Technology Co., Ltd. After filtering and merging raw reads, the optimized sequences were clustered into operational taxonomic units (OTUs) at 97% similarity. Alpha diversity, beta diversity, and microbial composition were analyzed using the Omicsmart platform.

### Statistical analysis

Microbial data were analyzed on the Majorbio Cloud platform.^1^ The Mothur software was used to calculate bacterial *α*-diversity(Chao index and Shannon index). The overall differences in bacterial communities among different samples were evaluated by PCoA based on Bray-Curtis distance combined with ANOSIM/Adonis methods. Further, Linear discriminant analysis effect size (LEfSe) was combined to screen out the species that had a greater impact on the differences between groups.

Other data were preprocessed in Microsoft Excel 2021. Normality and homogeneity of variances were assessed using Shapiro–Wilk and Levene’s tests, respectively. Data meeting assumptions were analyzed using one-way ANOVA, followed by Duncan’s multiple range test. Spearman’s correlation analysis was performed to assess associations between the relative abundances of the top 30 bacterial genera and host physiological parameters (including butyric acid concentration). Results were expressed as the mean ± standard error of the mean (SEM). Orthogonal polynomial contrasts were applied to test linear and quadratic trends. Statistical significance was set at *p* < 0.05. The ANOVA model used was *Y_ij_ = μ + τ_i_ + ε_ij_*, where *Y_ij_* is the dependent variable, *μ* is the overall mean, *τ_i_* is the treatment effect and *ε_ij_* is the random error. In the statistical model, the pen was considered as the experimental unit for all data (*N* = 6 replicates per treatment). For growth performance, diarrhea rate, and nutrient digestibility, data were calculated on a pen basis. For serum parameters, intestinal morphology, and microbiota composition, the values obtained from the single sampled pig per pen directly represented the respective pen replicate (*N* = 6).

## Results

### Growth performance of weaned piglets

The effects of dietary replacement of soybean meal with FP50 on growth performance are shown in Table 3. ADG, ADFI, F/G, and diarrhea rate were not affected by dietary FP50 inclusion during the experimental period. These results indicate that partial replacement of soybean meal with FP50 at levels up to 5% did not adversely affect growth performance or feed efficiency in weaned piglets.

**Table 3:** Growth performance of weaned piglets fed diets with graded levels of FP50.

Items	FP50 levels, %			SEM	*p*-value		
	0	3	5		ANOVA	Linear	Quadratic
IBW, kg	7.32	7.12	7.76	0.19	0.39	0.37	0.29
FBW, kg	18.25	18.41	20.32	0.53	0.24	0.13	0.44
ADFI, kg	0.62	0.65	0.74	0.03	0.26	0.13	0.62
ADG, kg	0.41	0.42	0.47	0.10	0.26	0.12	0.60
F/G, kg/kg	1.56	1.50	1.64	0.03	0.33	0.38	0.23
Diarrhea rate, %	12.95	10.83	11.62	1.11	0.76	0.64	0.58

IBW, initial body weight; FBW, final body weight; ADFI, average daily feed intake; ADG, average daily gain; SEM, standard error of the mean.

### Apparent total tract digestibility of nutrients

As shown in Table 4, dietary inclusion of FP50 improved nutrient utilization. The ATTD of DM and CP increased linearly with increasing levels of FP50 substitution (linear; *p* < 0.01). Ca digestibility also exhibited a positive linear response (linear; *p* < 0.05). The GE digestibility showed a quadratic trend of first slightly decreasing and then rising (quadratic; *p* < 0.01); the digestibility of Ash also showed a quadratic trend of first increasing and then decreasing (quadratic; *p* < 0.01). In contrast, the digestibility of EE, ADF, and P remained comparable among dietary treatments, whereas NDF digestibility showed a tendency toward improvement with increasing FP50 levels (*p* = 0.06).

**Table 4:** Apparent total tract digestibility of nutrients in weaned piglets fed diets with graded levels of FP50 (%).

Items	FP50 levels, %			SEM	*p*-value		
	0	3	5		ANOVA	Linear	Quadratic
DM	79.87^c^	81.45^b^	85.35^a^	0.58	< 0.01	< 0.01	< 0.01
CP	71.64^c^	75.20^b^	78.08^a^	0.77	< 0.01	< 0.01	0.73
EE	57.80	59.14	60.23	0.70	0.39	0.18	0.94
GE	82.83^b^	81.83^c^	86.51^a^	0.51	< 0.01	< 0.01	< 0.01
NDF	37.45	41.13	43.01	1.01	0.06	0.02	0.64
ADF	33.44	36.70	33.98	0.84	0.25	0.79	0.11
Ca	47.65^b^	51.79^ab^	54.10^a^	1.12	0.05	0.02	0.67
P	42.52	44.11	42.58	0.69	0.59	0.97	0.31
Ash	51.08^b^	62.29^a^	43.61^c^	1.93	< 0.01	< 0.01	< 0.01

DM, dry matter; CP, crude protein; EE, ether extract; GE, gross energy; NDF, neutral detergent fiber; ADF, acid detergent fiber; SEM, standard error of the mean.

Values within the same row with different superscript letters differ significantly. *p* < 0.05.

### Serum biochemical, immune, and antioxidant parameters

Serum CREA concentration increased linearly with FP50 inclusion (linear; *p* < 0.05; Table 5). Except for CREA, none of the other serum biochemical indices showed significant differences among the groups.

**Table 5:** Serum biochemical parameters of weaned piglets fed diets with graded levels of FP50.

Items	FP50 levels, %			SEM	*p*-value		
	0	3	5		ANOVA	Linear	Quadratic
TP, g/L	44.53	45.84	48.12	1.08	0.42	0.20	0.84
ALB, g/L	19.35	19.33	21.26	0.82	0.58	0.37	0.60
GLB, g/L	25.18	26.51	26.86	1.04	0.80	0.54	0.84
TC, mmol/L	2.27	2.54	2.47	0.08	0.35	0.30	0.31
TG, mmol/L	0.43	0.53	0.49	0.05	0.77	0.65	0.58
HDL, mmol/L	1.12	1.19	1.15	0.04	0.84	0.84	0.58
LDL, mmol/L	0.98	1.20	1.17	0.05	0.19	0.15	0.26
CREA, μmol/L	64.90^b^	73.00^ab^	75.29^a^	1.82	0.04	0.02	0.39
UREA, mmol/L	3.16	3.72	3.57	0.19	0.48	0.40	0.40
GLU, mmol/L	6.12	6.09	6.21	0.10	0.87	0.70	0.74
AST, U/L	61.17	59.95	52.05	3.10	0.46	0.25	0.62
ALT, U/L	90.18	87.18	89.69	4.95	0.97	0.97	0.82
ALP, U/L	375.20	322.65	309.79	16.85	0.22	0.10	0.60

TP, total protein; ALB, albumin; GLB, globulin; TC, total cholesterol; TG, triglyceride; HDL, high-density lipoprotein; LDL, low-density lipoprotein; CREA, creatinine; GLU, glucose; AST, aspartate aminotransferase; ALT, alanine aminotransferase; ALP, alkaline phosphatase; SEM, standard error of the mean.

Values within the same row with different superscript letters differ significantly. *p* < 0.05.

Serum immunoglobulin concentrations, including IgA, IgG, IgM, and SIgA, remained comparable among dietary treatments (Table 6), indicating that immune status was maintained throughout the experimental period.

**Table 6:** Serum immunoglobulin concentrations of weaned piglets fed diets with graded levels of FP50.

Items	FP50 levels, %			SEM	*p*-value		
	0	3	5		ANOVA	Linear	Quadratic
IgA, g/L	1.08	1.17	1.19	0.03	0.36	0.18	0.67
IgG, g/L	7.54	8.08	8.81	0.26	0.10	0.05	0.43
IgM, g/L	0.72	0.70	0.74	0.02	0.70	0.69	0.46
SIgA, mg/L	12.70	13.28	13.89	0.24	0.13	0.05	0.98

IgA, immunoglobulin A; IgG, immunoglobulin G; IgM, immunoglobulin M; SIgA, secretory immunoglobulin A; SEM, standard error of the mean.

Dietary FP50 inclusion modulated antioxidant-related indices (Table 7). SOD activity increased linearly with increasing FP50 levels (linear; *p* < 0.05), with the highest activity observed in the 5% FP50 group. T-AOC was quadratically affected by dietary treatment and reached its maximum value in the 3% FP50 group (quadratic; *p* < 0.05). CAT activity decreased linearly with increasing FP50 inclusion (linear; *p* < 0.05), whereas GSH-Px activity and MDA concentration remained stable across treatments.

**Table 7:** Serum antioxidant parameters of weaned piglets fed diets with graded levels of FP50.

Items	FP50 levels, %			SEM	*p*-value		
	0	3	5		ANOVA	Linear	Quadratic
SOD, U/mL	18.32^b^	19.53^b^	28.51^a^	1.66	0.01	0.01	0.19
GSH-Px, U/mL	1020.54	1137.30	1102.70	51.45	0.73	0.67	0.51
CAT, U/mL	137.05	124.41	107.58	5.58	0.09	0.03	0.85
T-AOC, U/mL	0.55^c^	0.66^a^	0.56^b^	0.02	0.05	0.86	0.02
MDA, nmol/mL	1.90	1.84	1.89	0.07	0.98	0.96	0.70

SOD, superoxide dismutase; GSH-Px, glutathione peroxidase; CAT, catalase; T-AOC, total antioxidant capacity; MDA, malondialdehyde; SEM, standard error of the mean.

Values within the same row with different superscript letters differ significantly. *p* < 0.05.

### Jejunal morphology

**Figure 1:**
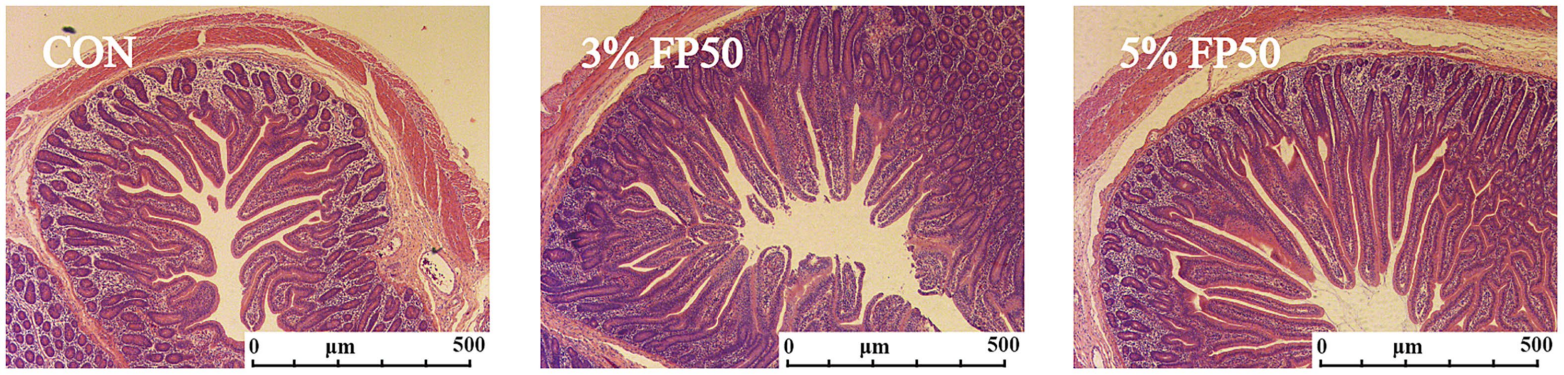
Representative photomicrographs of jejunal morphology (H&E staining, 40 × magnification) in weaned piglets with graded levels of FP50. H&E, hematoxylin and eosin.

Dietary FP50 supplementation improved jejunal morphology (Table 8). Specifically, CD decreased linearly (linear; *p* < 0.05) and the V/C ratio increased linearly (linear; *p* < 0.05) with increasing FP50 levels. VH tended to increase with increasing FP50 inclusion. Compared with the control group, dietary inclusion of FP50 resulted in more intact and orderly jejunal villus structures (Figure 1).

**Table 8:** Jejunal morphology of weaned piglets fed diets with graded levels of FP50.

Items	FP50 levels, %			SEM	*p*-value		
	0	3	5		ANOVA	Linear	Quadratic
VH, μm	469.71	477.14	516.14	11.15	0.19	0.09	0.50
CD, μm	160.87^a^	156.49^a^	151.19^b^	1.54	0.03	0.01	0.88
V/C	2.93^b^	3.04^b^	3.43^a^	0.08	0.02	0.01	0.38

VH, villus height; CD, crypt depth; SEM = standard error of the mean.

Values within the same row with different superscript letters differ significantly. *p* < 0.05.

### Cecal fermentation characteristics

As presented in Table 9, concentrations of major volatile fatty acids, including acetic, propionic, butyric, and valeric acids, were not affected by dietary FP50 inclusion. In contrast, concentrations of branched-chain fatty acids (BCFAs), including isobutyric acid and isovaleric acid, decreased linearly with increasing FP50 levels (linear; *p* < 0.05).

**Table 9:** Cecal volatile fatty acid concentrations of weaned piglets fed diets with graded levels of FP50 (μg/g).

Items	FP50 levels, %			SEM	*p*-value		
	0	3	5		ANOVA	Linear	Quadratic
Volatile fatty acid	11443.38	11091.98	11709.87	297.19	0.73	0.77	0.47
Acetic acid	6589.72	6356.21	6890.32	178.01	0.50	0.51	0.33
Propionic acid	2463.49	2430.09	2608.64	78.43	0.67	0.51	0.54
Isobutyric acid	153.29	118.66	102.87	9.44	0.08	0.03	0.61
Butyric acid	1634.71	1697.12	1845.62	109.92	0.75	0.47	0.86
Isovaleric acid	277.63	225.01	206.32	13.89	0.09	0.04	0.53
Valeric acid	324.55	264.89	277.90	12.13	0.09	0.09	0.14

SEM, standard error of the mean.

### Cecal microbiota composition

**Figure 2:**
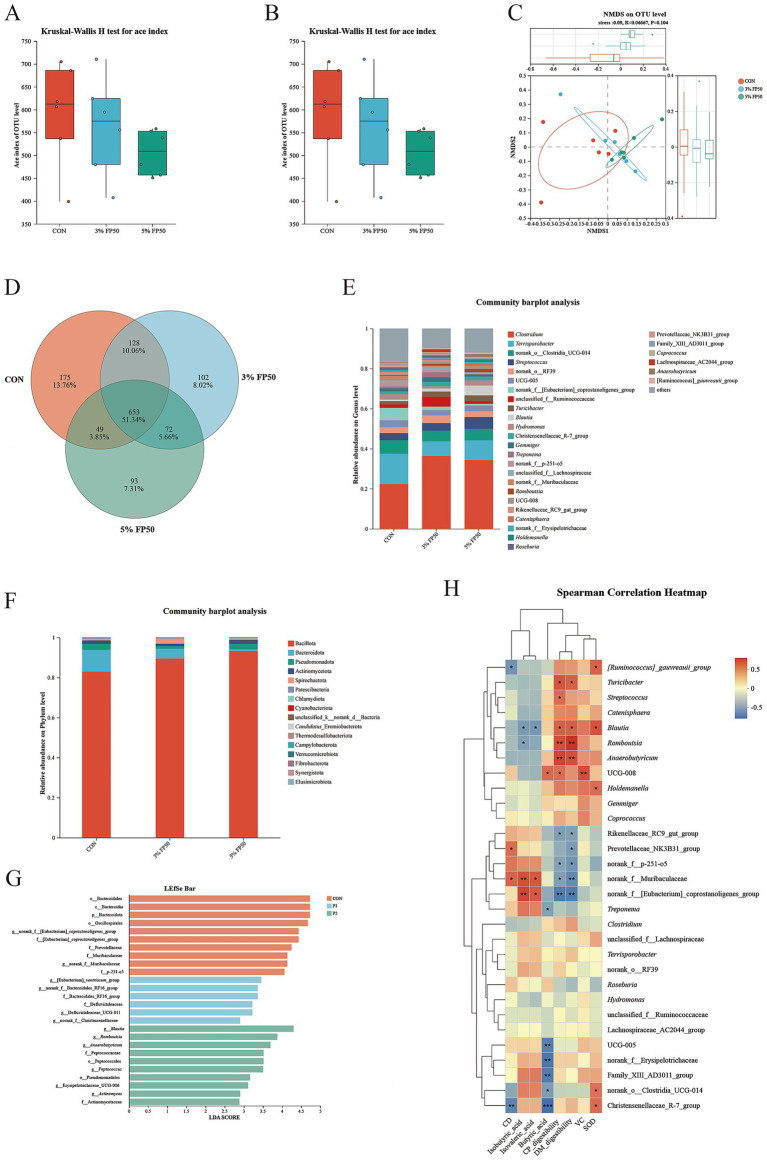
Effects of dietary FP50 levels on cecal microbial composition and Spearman correlations with host parameters in weaned piglets. **(A)** Shannon index. **(B)** Chao index. **(C)** NMDS analysis on OTU level. **(D)** Venn diagram of the OTUs. **(E)** Relative abundance of bacterial genera. **(F)** Relative abundance of bacterial phyla. **(G)** Differential bacterial genera identified by LEfSe analysis (LDA score > 2.0). **(H)** Spearman correlation heatmap between cecal bacterial genera and host physiological parameters. *p < 0.05, **p < 0.01, ***p < 0.001.

16S rRNA gene sequencing results indicated that the *α*-diversity indices of the cecal microbiota were similar among dietary treatments (Figures 2A,B). Non-metric multidimensional scaling (NMDS) analysis based on Bray–Curtis distance revealed no distinct separation of microbial community structure among groups, which was statistically confirmed by ANOSIM analysis (Figure 2C; stress = 0.09, *R* = 0.067, *p* = 0.104). A total of 51.34% of the operational taxonomic units (OTUs) were shared across all treatments, indicating the presence of a stable core microbiota (Figure 2D). At the genus level, *Clostridium*, *Terrisporobacter*, and norank_o__Clostridia_UCG-014 were the dominant taxa across all dietary treatments (Figure 2E), while *Bacillota* represented the predominant phylum (Figure 2F).

LEfSe analysis was performed to identify specific bacterial taxa that were differentially enriched among the groups (Figure 2G). The control group was characterized by the enrichment of *Bacteroidota*-associated taxa (e.g., class Bacteroidia, phylum *Bacteroidota*, and order Bacteroidales), norank_f__Muribaculaceae, and norank_f__[Eubacterium]_coprostanoligenes_group. The 3% FP50 group showed enrichment of norank_f__Bacteroidales_RF16_group, Bacteroidales_RF16_group, and norank_f__Christensenellaceae. In the 5% FP50 group, *Blautia*, *Romboutsia*, *Anaerobutyricum*, Peptococcaceae, and Peptococcales were significantly enriched.

To establish the potential links between the altered cecal microbiota and host physiological status, a Spearman correlation analysis was conducted between the top 30 bacterial genera and key host parameters (Figure 2H). Notably, the genera enriched in the 5% FP50 group—specifically *Blautia*, *Romboutsia*, and *Anaerobutyricum*—exhibited strong and consistent positive correlations (*p* < 0.05) with the apparent total tract digestibility (ATTD) of DM and CP, jejunal V/C ratio, and serum SOD activity, while displaying negative correlations (*p* < 0.05) with jejunal CD and cecal concentrations of branched-chain fatty acids (isobutyric and isovaleric acids). Conversely, the genera enriched in the control group, such as norank_f__Muribaculaceae and norank_f__[Eubacterium]_coprostanoligenes_group, were positively correlated with CD and BCFAs, but negatively correlated with nutrient digestibility (*p* < 0.05). Additionally, Christensenellaceae_R-7_group showed strong negative correlations with CD and BCFAs (*p* < 0.01).

## Discussion

Given the feed industry’s reliance on soybean meal and the growing pressure for sustainable resources (27), evaluating novel and efficient protein sources is critical. From a practical standpoint, maintaining normal growth performance is the primary prerequisite for evaluating the feasibility of any novel protein source in piglet feed (28–30). The present study systematically evaluated a commercially scalable *Candida utilis* SCP (FP50) produced through liquid fermentation using corn steep liquor as a substrate. Compared with many previous studies focusing on high substitution levels or additive effects of microbial proteins, the present work specifically examined the nutritional and physiological responses to low-level soybean meal substitution (3–5%) under practical feeding conditions.

In this study, replacing soybean meal with FP50 at 3% or 5% did not significantly affect ADG, ADFI, F/G, or diarrhea incidence, indicating that low-level FP50 substitution did not impair growth performance or intestinal tolerance under the present conditions. Although the 5% FP50 group showed a numerically higher final body weight than the control group, this difference was not statistically significant and may have been partly influenced by the numerically higher initial body weight in this group. Because growth performance was analyzed using the pen as the experimental unit with five replicate pens per treatment, the current design had limited power to detect moderate differences in growth traits. Therefore, larger-scale feeding trials are needed to determine whether this numerical difference represents a reproducible biological response. During the weaning period, the digestive and immune systems of piglets have not fully developed and matured, making them highly sensitive to changes in dietary protein sources (31). Previous studies have reported that when the proportion of SCP added is too high or the processing technology is improper, it may inhibit feed intake and growth performance due to reasons such as unsynchronized amino acid release and accumulation of hard-to-digest components in the cell wall (32–34). Therefore, the lack of negative impact on growth performance in this study may be due to the fact that the substitution level of FP50 is within a reasonable application range and the liquid fermentation process effectively improves its nutritional availability (35, 36).

In terms of nutrient digestion and utilization, FP50 significantly improved the apparent digestibility of DM and CP when substituted for soybean meal, and showed a linear increase with increasing substitution levels, indicating higher digestive and utilization efficiency when providing an equivalent amount of protein (37–39). Existing research has shown that *C. utilis* SCP processed by liquid fermentation typically contains a higher proportion of soluble proteins and small molecular peptides, which are more easily hydrolyzed and absorbed by digestive enzymes, thereby enhancing the utilization efficiency of amino acids in the foregut (40–42). The high nutrient digestibility observed in the present study may be partly associated with the intrinsic digestibility characteristics of FP50. *In vitro* evaluation showed that FP50 had crude protein digestibility of 96.05%, indicating that the yeast-derived protein was highly accessible to digestive enzymes. Furthermore, endogenous enzymes and metabolites released during the autolysis of yeast can further promote the digestion of other nutrients in the diet, thereby enhancing the overall nutrient utilization level of feed (43, 44). The simultaneous improvement in Ca apparent digestibility in this study also supports the potential role of FP50 in improving the intestinal digestion environment and promoting mineral absorption. However, the apparent digestibility of ash showed a non-linear and inconsistent response, with an increase at 3% FP50 but a decrease at 5% FP50. This result should be interpreted cautiously because ash digestibility reflects the apparent disappearance of total inorganic matter rather than the utilization of a specific mineral and can be influenced by dietary mineral composition, endogenous mineral losses, and marker-based analytical variation. In contrast to the inconsistent ash response, Ca digestibility increased linearly, whereas P digestibility remained unchanged. Therefore, the ash result cannot be taken as evidence of a consistent change in overall mineral utilization, and mineral-specific digestibility or balance studies are needed for further clarification.

The improvement in the morphological structure of the small intestine provides an important histological basis for enhancing digestibility. The results of this study show that as the substitution ratio of FP50 increases, the CD of the jejunum significantly decreases, while the V/C ratio significantly increases, indicating that intestinal epithelial cell turnover tends to stabilize and the effective absorption area expands. Weaning piglets often experience intestinal villus atrophy and crypt hyperplasia due to nutritional stress, thereby reducing digestive and absorptive efficiency (15, 45, 46). Previous studies have indicated that *β*-glucan and mannan in the yeast cell wall can mitigate the adverse effects of weaning stress on intestinal structure by regulating intestinal mucosal immune responses, enhancing tight junction protein expression, and improving intestinal barrier integrity (47, 48). Furthermore, mannan can block the adhesion of pathogenic bacteria to the intestinal mucosa, reduce the competitive consumption of dietary protein by pathogens, and enable the host to absorb and utilize more protein (49, 50). The simultaneous improvement in intestinal morphology and digestibility in this study suggests that FP50 can promote the function of nutrient absorption by maintaining the integrity of the intestinal structure.

With the increase of FP50 substitution level, a linear decrease in cecal BCFAs (such as isobutyric acid and isovaleric acid) was observed, accompanied by a linear increase in the ATTD of CP, which indicates that the substrate available for bacterial protein hydrolysis in the downstream gut was reduced. Since BCFAs are recognized markers of excessive or abnormal protein fermentation in the large intestine, their decline implies the improvement of protein digestion and absorption in the foregut, thereby limiting the entry of undigested nitrogen into the cecum (51, 52). Crucially, Spearman correlation analysis showed that the genera significantly enriched in the 5% FP50 group (such as *Blautia*, *Romboutsia*, and *Anaerobutyricum*) showed strong positive correlations (*p* < 0.05) with key indicators of nutrient utilization and intestinal integrity, including the ATTD of DM and CP, V/C, and SOD activity. Conversely, these genera showed significant negative correlations (*p* < 0.05) with markers of intestinal stress and inefficient protein fermentation, namely jejunal CD and cecal BCFAs concentration. This pattern is highly consistent with the known predicted functional roles of these taxa. Previous studies have shown that *Blautia* and *Romboutsia* are butyrate-producing bacteria (53, 54), and butyrate is the main energy source for colon epithelial cells, which is essential for maintaining the intestinal epithelial barrier and inhibiting inflammation (55, 56). The negative correlation between these butyrate-producing genera and BCFAs further supports the hypothesis that a shift towards carbohydrate fermentation (and away from protein hydrolysis) is occurring. At the phylum level, the increase in the relative abundance of *Bacillota* and the decrease in the proportion of *Bacteroidota* led to a higher *Bacillota/Bacteroidota* ratio, which is consistent with the observations in studies reporting improved nutrient utilization efficiency (57, 58). The stability of *α*-diversity indices and the lack of distinct separation in *β*-diversity indicate that FP50 does not cause broad disruption of the microbial community. Instead, these changes are highly specific and function-oriented, as evidenced by the enrichment of taxa with specific metabolic capabilities (such as butyrate production) and their direct statistical association with beneficial host outcomes. This suggests that FP50 exerts its positive effects on gut health primarily by reshaping the metabolic potential of the cecal microbiota rather than by altering its overall taxonomic composition. It should be acknowledged that direct measurements of ileal CP digestibility and other nitrogenous metabolites (such as ammonia, indoles, and phenols) were not performed in this study. Therefore, the proposed link between enhanced foregut digestibility and reduced hindgut protein hydrolysis fermentation remains a highly plausible hypothesis, strongly supported by convergent evidence from BCFAs, ATTD, and the microbial-host correlation network. Future studies using piglet fistula models and targeted metabolomics analysis of nitrogenous compounds are needed to clearly confirm this metabolic pathway.

The optimization of the hindgut fermentation profile and the improvement of jejunal morphology are often associated with a reduction in systemic oxidative stress, as a healthier gut barrier limits the translocation of luminal endotoxins and pro-inflammatory metabolites into the circulation (59, 60). In the present study, dietary FP50 inclusion altered several serum antioxidant-related indices, although these responses were not uniformly consistent across all measured markers. Circulating SOD activity increased linearly with increasing FP50 levels, which suggests an upregulation of the enzymatic defense system against superoxide radicals (61, 62). However, this increase in SOD was not accompanied by a reduction in MDA concentration—a primary biomarker of lipid peroxidation—and CAT activity was linearly decreased, while GSH-Px activity and T-AOC remained largely unaffected. These inconsistent findings suggest that while FP50 modulated specific components of the systemic antioxidant defense system, it did not elicit a generalized enhancement of systemic antioxidant capacity. The decrease in CAT activity, in contrast to the increase in SOD, might reflect a feedback regulation or a localized shift in hydrogen peroxide metabolism that warrants further investigation. Therefore, the antioxidant benefits of FP50 should be interpreted cautiously, and further studies utilizing a wider range of redox biomarkers and tissue-specific analyses are required to clarify the precise impact of FP50 on the redox status of piglets.

Beyond systemic redox status, the stability of serum biochemical parameters serves as a critical indicator for evaluating the metabolic safety and tolerance of novel protein sources in animal diets. In this study, the concentrations of serum parameters related to liver function, lipid metabolism, and glucose remained stable across all treatments, supporting the metabolic safety of FP50 as a partial replacement for soybean meal. However, serum CREA concentration exhibited a linear increase with increasing FP50 inclusion levels. Because elevated serum creatinine is traditionally associated with impaired glomerular filtration, this finding must be interpreted with caution. In the present study, the linear increase in CREA was not accompanied by any elevation in serum UREA—another key indicator of renal clearance—nor did it affect liver- or tissue-damage enzymes. Furthermore, the serum CREA values in all groups (64.90 to 75.29 μmol/L) remained well within the normal physiological range for healthy weaned piglets (50 to 110 μmol/L) (63). Thus, the isolated increase in CREA does not provide sufficient evidence of renal dysfunction under the current experimental conditions. Alternatively, serum creatinine is a metabolic byproduct of muscle creatine and phosphocreatine, and its circulating level is positively correlated with skeletal muscle mass, nitrogen retention, and active protein turnover in growing pigs (64, 65). This physiological explanation aligns well with the linear improvements in the apparent total tract digestibility of DM and CP observed in Table 4. The highly digestible protein and peptide fractions in FP50 likely enhanced amino acid availability in the foregut, thereby supporting muscle protein synthesis and systemic nitrogen utilization (15). Nevertheless, because muscle mass and nitrogen balance were not directly measured in this trial, this metabolic explanation remains speculative, and further studies incorporating renal clearance markers are needed to fully elucidate the biological significance of the elevated CREA.

It should be noted that in the results of this study, although multiple indicators exhibited a consistent and biologically meaningful trend of improvement, some parameters did not reach statistical significance, which may be related to factors such as the short duration of the trial and significant individual differences. Furthermore, changes in gut microbiota and antioxidant status are more reflected in functional regulation, and their underlying molecular mechanisms still need to be further elucidated through transcriptomics, metabolomics, or signaling pathway research.

## Conclusion

In conclusion, liquid-fermentation-derived *C. utilis* SCP (FP50) serves as a highly effective alternative to soybean meal (3–5%) in weaned piglet diets. The nutritional benefit of FP50 extends beyond simple protein substitution; it optimizes the intestinal environment by enhancing protein disappearance in the foregut. The improved nutrient digestibility and jejunal morphology, coupled with the reduction in hindgut branched-chain fatty acids (BCFAs), suggest that FP50 mitigates the risk of proteolytic fermentation. Crucially, these physiological improvements were strongly correlated with the enrichment of specific cecal genera (such as *Blautia*, *Romboutsia*, and *Anaerobutyricum*), highlighting a coordinated host-microbiota response. These findings provide a physiological basis for using liquid-fermented microbial proteins to maintain gut homeostasis and support sustainable pig production through soybean meal reduction.

## Data Availability

The data presented in the study are deposited in the NCBI BioProject repository, accession number PRJNA1477322.

## Footnotes

1
https://cloud.majorbio.com

## References

[1] Verstraete W Clauwaert P Vlaeminck SE. Used water and nutrients: recovery perspectives in a ‘panta rhei’ context. Bioresour Technol. (2016) 215:199–208. doi: 10.1016/j.biortech.2016.04.094, 27184651

[2] Esteves EA Martino HSD Oliveira FCE Bressan J Costa NMB. Chemical composition of a soybean cultivar lacking lipoxygenases (LOX_2_ and LOX_3_). Food Chem. (2010) 122:238–42. doi: 10.1016/j.foodchem.2010.02.069

[3] Berners-Lee M Kennelly C Watson R Hewitt CN. Current global food production is sufficient to meet human nutritional needs in 2050 provided there is radical societal adaptation. Elementa. (2018) 6:52. doi: 10.1525/elementa.310, 33021500

[4] Tao A Wang J Luo B Liu B Wang Z Chen X . Research progress on cottonseed meal as a protein source in pig nutrition: an updated review. Animal. Nutrition. (2024) 18:220–33. doi: 10.1016/j.aninu.2024.03.020, 39281049 PMC11402386

[5] Tang Q Lan T Zhou C Gao J Wu L Wei H . Nutrition strategies to control post-weaning diarrhea of piglets: from the perspective of feeds. Animal. Nutrition. (2024) 17:297–311. doi: 10.1016/j.aninu.2024.03.006, 38800731 PMC11127239

[6] Zhao M-Q Chen X Chen Z-M Liu G-H Zheng A-J. Research progress on anti-nutritional factors in soybean meal and preparation of peptides from enzymatically hydrolyzed soybean meal. China Anim Husband Vet Med. (2024) 51:1931–8. doi: 10.16431/j.cnki.1671-7236.2024.05.014

[7] Ma SF Wang H Dou YL Liang XF Zheng YH Wu XF . Anti-nutritional factors and protein dispersibility index as principal quality indicators for soybean meal in diet of Nile Tilapia (*Oreochromis niloticus* GIFT), a Meta-analysis. Animals. (2022) 12:1831. doi: 10.3390/ani12141831, 35883378 PMC9312040

[8] Kujan P Prell A Šafář H Sobotka M Řezanka T Holler P. Use of the industrial yeast *Candida utilis* for cadmium sorption. Folia Microbiol. (2006) 51:257–60. doi: 10.1007/BF02931807, 17007420

[9] Rehman AU Rasool S Mukhtar H Haq IU. Production of an extracellular lipase by *Candida utilis* NRRL-Y-900 using agro-industrial by-products. Turk J Biochem. (2014) 39:140–9. doi: 10.5505/tjb.2014.96977

[10] Nie Q . The strains and functions of the yeast-based biological feed. Chinese Feed. (2018) 607:89–93.

[11] Zhang X Zhao G. Yeast cultivation for single-cell protein production using the carbohydrate hydrolysate of steam-exploded Eucalyptus wood. Wood Research. (2022) 67:568–81. doi: 10.37763/wr.1336-4561/67.4.568581

[12] Zhao G Zhang W Zhang G. Production of single cell protein using waste capsicum powder produced during capsanthin extraction. Lett Appl Microbiol. (2010) 50:187–91. doi: 10.1111/j.1472-765X.2009.02773.x, 20002572

[13] Qi HY . Research progress on *candida utilis* protein feed. Chinese. J Vet Med. (2023) 43:844–8. doi: 10.16303/j.cnki.1005-4545.2023.04.30

[14] Wu PY . Differences in acid stress response of Lacticaseibacillus paracasei Zhang cultured from solid-state fermentation and liquid-state fermentation. Microorganisms. (2021) 9. doi: 10.3390/microorganisms9091951, 34576848 PMC8465097

[15] Cruz A Håkenåsen IM Skugor A Mydland LT Åkesson CP Hellestveit SS . *Candida utilis* yeast as a protein source for weaned piglets: effects on growth performance and digestive function. Livest Sci. (2019) 226:31–9. doi: 10.1016/j.livsci.2019.06.003

[16] Zeng Y Yin H Zhou X Wang C Zhou B Wang B . Effect of replacing inorganic iron with iron-rich microbial preparations on growth performance, serum parameters and iron metabolism of weaned piglets. Vet Res Commun. (2023) 47:2017–25. doi: 10.1007/s11259-023-10162-6, 37402083

[17] Sahlmann C Djordjevic B Lagos L Mydland LT Morales-Lange B Øvrum Hansen J . Yeast as a protein source during smoltification of Atlantic salmon (*Salmo salar* L.), enhances performance and modulates health. Aauaculture. (2019) 513:734396. doi: 10.1016/j.aquaculture.2019.734396

[18] Overland M . Evaluation of *Candida utilis*, Kluyveromyces marxianus and *Saccharomyces cerevisiae* yeasts as protein sources in diets for Atlantic salmon (*Salmo salar*). Aquaculture. (2013) 402-403:1–7. doi: 10.1016/j.aquaculture.2013.03.016, 38826717

[19] Qi HY . Integrated microbiome and metabolomics analysis of the effects of dietary supplementation with corn-steep-liquor-derived *candida utilis* feed on black pigs. Animals. (2024) 14:306. doi: 10.3390/ani14020306, 38254475 PMC10812819

[20] Da Silva TAF . Cost-effective fibrinolytic enzyme production by microalga *Dunaliella tertiolecta* using medium supplemented with corn steep liquor. An Acad Bras Cienc. (2023) 95:e20220552. doi: 10.1590/0001-3765202320220552, 37585969

[21] Zhao F Zhang HF Zhang ZY. Monogastric animal bionic digestion system operation manual. Chin Acad Agric Sci. (2011)

[22] NRC. Nutrient Requirements of Swine. 11th Rev. ed. Washington, DC: The National Academies Press (2012).

[23] Sun HY Kim IH. Effect of yeast culture (*Saccharomyces cerevisiae*) and garlic (*Allium sativum*) product mixture on growth performance, nutrient digestibility, faecal microflora, faecal noxious-gas emission and meat quality in finishing pigs. Animal Prouduction. Science. (2020) 60:1911–7. doi: 10.1071/AN18722, 38477348

[24] Petersen SO Højberg O Poulsen M Schwab C Eriksen J. Methanogenic community changes, and emissions of methane and other gases, during storage of acidified and untreated pig slurry. J Appl Microbiol. (2014) 117:160–72. doi: 10.1111/jam.12498, 24636626

[25] Stevens RJ Laughlin RJ Frost JP. Effect of acidification with sulphuric acid on the volatilization of ammonia from cow and pig slurries. J Agric Sci. (1989) 113:389–95. doi: 10.1017/S0021859600070106

[26] Scott TA Hall JW. Using acid insoluble ash marker ratios (diet:digesta) to predict digestibility of wheat and barley metabolizable energy and nitrogen retention in broiler chicks. Poult Sci. (1998) 77:674–9. doi: 10.1093/ps/77.5.674, 9603354

[27] Hafez HM Attia YA. Challenges to the poultry industry: current perspectives and strategic future after the COVID-19 outbreak. Front Vet Sci. (2020) 7:516. doi: 10.3389/fvets.2020.00516, 33005639 PMC7479178

[28] Zhang S Wu Z Heng J Song H Tian M Chen F . Combined yeast culture and organic selenium supplementation during late gestation and lactation improve preweaning piglet performance by enhancing the antioxidant capacity and milk content in nutrient-restricted sows. Animal. Nutrition. (2020) 6:160–7. doi: 10.1016/j.aninu.2020.01.004, 32542196 PMC7283508

[29] Lv L Zhang H Liu Z Lei L Feng Z Zhang D . Comparative study of yeast selenium vs. sodium selenite on growth performance, nutrient digestibility, anti-inflammatory and anti-oxidative activity in weaned piglets challenged by Salmonella typhimurium. Innate Immun. (2020) 26:248–58. doi: 10.1177/1753425919888566, 31766926 PMC7251790

[30] Jiayu M . The effects of *Candida utilis* on the growth performance, serum immunity and antioxidant indices, as well as the apparent nutrient digestibility of weaned piglets. Journal of animal. Nutrition. (2022) 34:2260–71. doi: 10.3969/j.issn.1006-267x.2022.04.022

[31] Upadhaya SD Kim IH. The impact of weaning stress on gut health and the mechanistic aspects of several feed additives contributing to improved gut health function in weanling piglets-a review. Animals (Basel). (2021) 11:2418. doi: 10.3390/ani11082418, 34438875 PMC8388735

[32] Rodríguez B . Evaluation of torula yeast (*Candida utilis*) grown on distillery vinasse for broilers. Cuban J Agric Sci. (2013) 47:183–8.

[33] Cruz A Sterten H Steinhoff FS Mydland LT Øverland M. Cyberlindnera jadinii yeast as a protein source for broiler chickens: effects on growth performance and digestive function from hatching to 30 days of age. Poult Sci. (2020) 99:3168–78. doi: 10.1016/j.psj.2020.01.023, 32475453 PMC7597667

[34] Pourelmi M . Evaluation of single cell protein as a non-conventional feedstuff in broilers feeding. Iran J Appl Anim Sci. (2018) 8:317–24.

[35] Hombegowda GP Suresh BN Shivakumar MC Ravikumar P Girish BC Rudrappa SM . Growth performance, carcass traits and gut health of broiler chickens fed diets incorporated with single cell protein. Animal. Bioscience. (2021) 34:1951–62. doi: 10.5713/ab.20.0844, 33902179 PMC8563244

[36] Boontiam W Bunchasak C Kim YY Kitipongpysan S Hong J. Hydrolyzed yeast supplementation to newly weaned piglets: growth performance, gut health, and microbial fermentation. Animals (Basel). (2022) 12:350. doi: 10.3390/ani12030350, 35158673 PMC8833445

[37] Huang B Shi M Pang A Tan B Xie S. Effects of fishmeal replacement by clostridium autoethanogenum protein meal on cholesterol bile acid metabolism, antioxidant capacity, hepatic and intestinal health of pearl gentian grouper (*epinephelus fuscoguttatus* ♀ × *epinephelus lanceolatus* ♂). Animals (Basel). (2023) 13:1090. doi: 10.3390/ani13061090, 36978631 PMC10044235

[38] Zhu SJ . Partial substitution of fish meal by Clostridium autoethanogenum protein in the diets of juvenile largemouth bass (*Micropterus salmoides*). Aquaculture Rrports. (2022) 22:100938. doi: 10.1016/j.aqrep.2021.100938, 38826717

[39] Reilly P O’Doherty JV Pierce KM Callan JJ O’Sullivan JT Sweeney T. The effects of seaweed extract inclusion on gut morphology, selected intestinal microbiota, nutrient digestibility, volatile fatty acid concentrations and the immune status of the weaned pig. Animal. (2008) 2:1465–73. doi: 10.1017/S1751731108002711, 22443904

[40] Jach ME Serefko A Ziaja M Kieliszek M. Yeast protein as an easily accessible food source. Metabolites. (2022) 12:63. doi: 10.3390/metabo12010063, 35050185 PMC8780597

[41] Bakory MTA. Yeast culture in animal nutrition: a review. Biosci Res. (2014) 11:10–9.

[42] Yamada EA Sgarbieri VC. Yeast (*Saccharomyces cerevisiae*) protein concentrate: preparation, chemical composition, and nutritional and functional properties. J Agric Food Chem. (2005) 53:3931–6. doi: 10.1021/jf0400821, 15884819

[43] Overland M Schoyen HF Skrede A. Growth performance and carcase quality in broiler chickens fed on bacterial protein grown on natural gas. Br Poult Sci. (2010) 51:686–95. doi: 10.1080/00071668.2010.522556, 21058073

[44] Latorre JD Hernandez-Velasco X Vicente JL Wolfenden R Hargis BM Tellez G. Effects of the inclusion of a Bacillus direct-fed microbial on performance parameters, bone quality, recovered gut microflora, and intestinal morphology in broilers consuming a grower diet containing corn distillers dried grains with solubles. Poult Sci. (2017) 96:2728–35. doi: 10.3382/ps/pex082, 28419329 PMC5850462

[45] Qin L Ji W Wang J Li B Hu J Wu X. Effects of dietary supplementation with yeast glycoprotein on growth performance, intestinal mucosal morphology, immune response and colonic microbiota in weaned piglets. Food Funct. (2019) 10:2359–71. doi: 10.1039/c8fo02327a, 30972390

[46] Furbeyre H van Milgen J Mener T Gloaguen M Labussière E. Effects of dietary supplementation with freshwater microalgae on growth performance, nutrient digestibility and gut health in weaned piglets. Animal. (2017) 11:183–92. doi: 10.1017/S1751731116001543, 27452961

[47] Yao K., The effects of ethanol Clostridium protein replacing soybean meal on the growth performance, immune function and intestinal health of weaned piglets 2023, Huazhong Agricultural University. doi: 10.27158/d.cnki.ghznu.2023.001989

[48] LlN XQ . The effects of ethanol bacillus protein on the growth performance, immune function and intestinal health of yellow-feathered broilers journal of animal. Nutrition. (2023) 35:3617–30. doi: 10.12418/CJAN2023.337

[49] Refstie S Baeverfjord G Seim RR Elvebø O. Effects of dietary yeast cell wall β-glucans and MOS on performance, gut health, and salmon lice resistance in Atlantic salmon (*Salmo salar*) fed sunflower and soybean meal. Aquaculture. (2010) 305:109–16. doi: 10.1016/j.aquaculture.2010.04.005

[50] Kogan G Kocher A. Role of yeast cell wall polysaccharides in pig nutrition and health protection. Livest Sci. (2007) 109:161–5. doi: 10.1016/j.livsci.2007.01.134

[51] Xin H Ma T Xu Y Chen G Chen Y Villot C . Characterization of fecal branched-chain fatty acid profiles and their associations with fecal microbiota in diarrheic and healthy dairy calves. J Dairy Sci. (2021) 104:2290–301. doi: 10.3168/jds.2020-18825, 33358167

[52] Li Z Ding L Zhu W Hang S. Effects of the increased protein level in small intestine on the colonic microbiota, inflammation and barrier function in growing pigs. BMC Microbiol. (2022) 22:172. doi: 10.1186/s12866-022-02498-x, 35794527 PMC9258065

[53] Li R Hou G Jiang X Song Z Fan Z Hou DX . Different dietary protein sources in low protein diets regulate colonic microbiota and barrier function in a piglet model. Food Funct. (2019) 10:6417–28. doi: 10.1039/c9fo01154d, 31517363

[54] Sun W Chen W Meng K Cai L Li G Li X . Dietary supplementation with probiotic *Bacillus licheniformis* S6 improves intestinal integrity via modulating intestinal barrier function and microbial diversity in weaned piglets. Biology (Basel). (2023) 12:238. doi: 10.3390/biology12020238, 36829515 PMC9953057

[55] Koh A de Vadder F Kovatcheva-Datchary P Bäckhed F. From dietary Fiber to host physiology: short-chain fatty acids as key bacterial metabolites. Cell. (2016) 165:1332–45. doi: 10.1016/j.cell.2016.05.041, 27259147

[56] Tan J McKenzie C Potamitis M Thorburn AN Mackay CR Macia L. The role of short-chain fatty acids in health and disease. Adv Immunol. (2014) 121:91–119. doi: 10.1016/b978-0-12-800100-4.00003-9, 24388214

[57] Sun X Cui Y Su Y Gao Z Diao X Li J . Dietary Fiber ameliorates lipopolysaccharide-induced intestinal barrier function damage in piglets by modulation of intestinal microbiome. mSystems. (2021) 6:e01374–20. doi: 10.1128/mSystems.01374-20, 33824201 PMC8547013

[58] Wang L Gao W Shi H Hu Q Lai C. Effects of replacing fishmeal and soybean protein concentrate with Degossypolized cottonseed protein in diets on growth performance, nutrient digestibility, intestinal morphology, cecum microbiome and fermentation of weaned piglets. Animals (Basel). (2022) 12:1667. doi: 10.3390/ani12131667, 35804565 PMC9264811

[59] Wyss M Kaddurah-Daouk R. Creatine and creatinine metabolism. Physiol Rev. (2000) 80:1107–213. doi: 10.1152/physrev.2000.80.3.1107, 10893433

[60] Caprarulo V Hejna M Giromini C Liu Y Dell’Anno M Sotira S . Evaluation of dietary Administration of Chestnut and Quebracho Tannins on growth, serum metabolites and Fecal parameters of weaned piglets. Animals (Basel). (2020) 10:1945. doi: 10.3390/ani10111945, 33105748 PMC7690424

[61] Liu L Chen D Yu B Luo Y Huang Z Zheng P . Influences of selenium-enriched yeast on growth performance, immune function, and antioxidant capacity in weaned pigs exposure to oxidative stress. Biomed Res Int. (2021) 2021:5533210. doi: 10.1155/2021/5533210, 33855070 PMC8019624

[62] Li MY . Diet supplemented with a novel Clostridium autoethanogenum protein have a positive effect on the growth performance, antioxidant status and immunity in juvenile Jian carp (*Cyprinus carpio* var Jian). Aquacult Rep. (2021) 19:100572. doi: 10.1016/j.aqrep.2020.100572, 38826717

[63] Metallo BF da Selva LCS da Fonseca AD Niblett RT Aviles-Rosa EO. The effect of weaning age on physiological, behavioral, and performance indicators of welfare in weaned piglets. Livest Sci. (2025) 301:105816. doi: 10.1016/j.livsci.2025.105816

[64] Kim H Shin H Kim YY. Effects of different levels of dietary crude protein on growth performance, blood profiles, diarrhea incidence, nutrient digestibility, and odor emission in weaning pigs. Animal. Bioscience. (2023) 36:1228–40. doi: 10.5713/ab.22.0440, 36915927 PMC10330979

[65] Millet S Aluwé M de Boever J de Witte B Douidah L van den Broeke A . The effect of crude protein reduction on performance and nitrogen metabolism in piglets (four to nine weeks of age) fed two dietary lysine levels. J Anim Sci. (2018) 96:3824–36. doi: 10.1093/jas/sky254, 29939350 PMC6127758

