# Supplementary material for: Liquid-fermentation-derived Candida utilis protein improves nutrient digestibility and intestinal health in weaned piglets
Source: Front Vet Sci. 2026 Jun 30;13:1862132. doi: 10.3389/fvets.2026.1862132 (PMC13364533; doi:10.3389/fvets.2026.1862132)
Supplement: Supplementary file 1 [file Table_1.DOCX]

**Table S1.** **Mycotoxin concentrations of FP50 (as-fed basis).**

| **Mycotoxins** | **Detection Results** | **Limit of Quantification** | **Detection Method** |
| --- | --- | --- | --- |
| T-2 toxin, μg/kg | not detected | 2.00 | NY/T 2071-2011 |
| Aflatoxin B1, μg/kg | not detected | 2.00 | NY/T 2071-2011 |
| Deoxynivalenol, μg/kg | not detected | 0.10 | GB/T 30956-2014 |
| Zearalenone^1^, μg/kg | 94.3 | 10.0 | NY/T 2071-2011 |
| Fumonisin, mg/kg | not detected | 0.05 | NY/T 1970-2010 4 |
| Fumonisin B₁, mg/kg | not detected | 0.05 | NY/T 1970-2010 4 |
| Fumonisin B₂, mg/kg | not detected | 0.05 | NY/T 1970-2010 4 |

^1^ The detected level of zearalenone was below the maximum limits recommended for feed ingredients.

**Table S2. In vitro digestibility and energy utilization of FP50 (as-fed basis).**

| **Items** | **Content** |
| --- | --- |
| DMD, % | 91.49 |
| CPD, % | 96.05 |
| GED, % | 96.38 |
| EHGE, MJ/kg | 15.47 |

DMD = In vitro dry matter digestibility; CPD = In vitro protein digestibility; GED = In vitro energy digestibility; EHGE = In vitro enzymatically hydrolyzed product energy value.
